# 4-Methoxycarbonyl Curcumin: A Unique Inhibitor of Both Inflammatory Mediators and Periodontal Inflammation

**DOI:** 10.1155/2013/329740

**Published:** 2013-12-24

**Authors:** Ying Gu, Hsi-Ming Lee, Nicole Napolitano, McKenzie Clemens, Yazhou Zhang, Timo Sorsa, Yu Zhang, Francis Johnson, Lorne M. Golub

**Affiliations:** ^1^Department of General Dentistry, School of Dental Medicine, Stony Brook University, Stony Brook, NY 11794-8706, USA; ^2^Department of Oral Biology and Pathology, School of Dental Medicine, Stony Brook University, Stony Brook, NY 11794, USA; ^3^Department of Oral and Maxillofacial Diseases, Institute of Dentistry, Helsinki University Central Hospital, University of Helsinki, Helsinki, Finland; ^4^Department of Chemistry, Stony Brook University, Stony Brook, NY 11794-3400, USA; ^5^Department of Pharmacological Sciences, Stony Brook University, Stony Brook, NY 11794, USA

## Abstract

Chronic inflammatory diseases such as periodontitis have been associated with increased risk for various medical conditions including diabetes and cardiovascular disease. Endotoxin (lipopolysaccharide, LPS), derived from gram-negative periodonto-pathogens, can induce the local accumulation of mononuclear cells in the inflammatory lesion, increasing proinflammatory cytokines and matrix metalloproteinases (MMPs). This ultimately results in the destruction of periodontal connective tissues including alveolar bone. Curcumin is the principal dyestuff in the popular Indian spice turmeric and has significant regulatory effects on inflammatory mediators but is characterized by poor solubility and low bioactivity. Recently, we developed a series of chemically modified curcumins (CMCs) with increased solubility and zinc-binding activity, while retaining, or further enhancing, their therapeutic effects. In the current study, we demonstrate that a novel CMC (CMC 2.5: 4-methoxycarbonyl curcumin) has significant inhibitory effects, better than the parent compound curcumin, on proinflammatory cytokines and MMPs in *in vitro*, in cell culture, and in an animal model of periodontal inflammation. The therapeutic potential of CMC 2.5 and its congeners may help to prevent tissue damage during various chronic inflammatory diseases including periodontitis and may reduce the risks of systemic diseases associated with this local disorder.

## 1. Introduction

Periodontal disease is one of the most common chronic inflammatory diseases encountered in humans. During the pathogenesis of this condition, anaerobic gram-negative periodontal-associated pathogens (e.g., *P. gingivalis, T. forsythia*) and the lipopolysaccharide (LPS, endotoxin) in their cell walls stimulate the innate and adaptive immune responses in periodontal tissues [1]. Inflammatory cells such as neutrophils and monocytes/macrophages are recruited to the lesion site and generate elevated levels of cytokines and other proinflammatory mediators such as the prostaglandins. The resulting periodontal inflammation upregulates matrix metalloproteinase (MMP) expression and, the activity of the latter, contributes to the destruction and loss of periodontal connective tissues including bone [2].

Curcumin [1,7-bis-(4-hydroxy-3-methoxyphenyl)-1,6-heptadiene-3,5-dione] is a component of the popular Indian spice turmeric and has been recommended for numerous medical applications [3]. Extensive investigations have led to the conclusion that it is a highly pleiotropic molecule with significant beneficial effects on inflammatory and other diseases including cancers such as multiple myeloma [3–5]. This natural product has long been used as an herbal anti-inflammatory treatment to relieve pain and inflammation in the skin and muscles and, for a variety of pulmonary, gastrointestinal and liver diseases, as well as a remedy for nonhealing wounds. These therapeutic effects have been studied in both *in vitro *and *in vivo* model systems [6–8]. Despite these many beneficial effects, curcumin has major limitations including poor solubility, a lack of systemic bioavailability, and rapid metabolic disposition [9]. Thus, extremely high oral doses of the compound are needed and, even then, it results in only very low levels in the systemic circulation of both animals and humans. This has severely limited its clinical application [10]. Recently, our laboratory has developed a series of novel chemically modified curcumins with a carbonyl substituent at the C-4 position [11, 12]. Such analogues have an additional electron-withdrawing group which enhances their anti-inflammatory therapeutic effects. One such compound (CMC 2.5) contains a methoxycarbonyl group at C4, shows an improved solubility, better serum albumin-binding activity, and greater acidity, and enhanced zinc-binding characteristics. This modification has been found to enhance the MMP-inhibitory properties of this novel compound versus curcumin [11, 12].

In the current report, we investigate the effect of this novel substance, 4-methoxycarbonylcurcumin (CMC 2.5), on proinflammatory cytokines and MMPs in an *in vivo* diabetes-enhanced periodontal inflammation rat model and in a relevant cell culture model. Rats with experimental diabetes mellitus manifest increased gingival inflammation and periodontal tissue destruction including alveolar bone loss [13–16]. This animal model of STZ-induced diabetes as an enhancer of periodontal disease is well established in our laboratory [14, 15] and has been described by others as well [16]. It was used in preclinical studies during the development of Periostat, the only host modulation and MMP inhibitory therapy for periodontitis approved by the FDA. This unique animal model, different from traditional rat models of experimental periodontitis using ligatures or oral pathogen infection, will allow us to study the possible association between this local inflammatory disease and relevant systemic conditions. We have previously demonstrated that the diabetic condition increases the levels of cytokines and MMPs locally in the gingival tissues as well as systemically in plasma [14, 15]. In addition, levels of MMP-8 in skin and both local and systemic bone loss were increased in this animal model [17]. Therefore, this will allow us to study not only the periodontal disease, but also the systemic factors associated with this local inflammatory condition.

In addition, a periodontal disease-relevant cell culture system involving human mononuclear cells challenged with LPS (derived from the periodontal pathogen, *P. gingivalis*) was also used to evaluate the effect of CMC 2.5 on the induced excessive levels of proinflammatory MMPs and cytokines. Nuclear factor-kappaB (NF-*κ*B) is a transcription factor involved in the cell signaling transduction pathway associated with inflammation and plays a key role in regulating the cellular immune response to stimuli such as stress, cytokines, and bacterial or viral antigens [18]. Dysregulation of NF-*κ*B has been linked to cancer and inflammatory diseases [18]. Therefore, the effect of CMC 2.5 on the activation/phosphorylation of NF-*κ*B was evaluated as well.

## 2. Materials and Methods

### 2.1. Chemical Reagents

All chemical reagents, LPS from *P. gingivalis* and curcumin, were purchased from Sigma-Aldrich Co. (St. Louis, MO). All cell culture reagents were purchased from Gibco/Invitrogen Corp. (Carlsbad, CA). CMC 2.5 was synthesized, purified (99.5% pure), and provided by Chem Master Intl. Inc., Stony Brook, NY.

### 2.2. *In Vitro* MMP Inhibition Assay (IC_50_)

Human chromatographically pure MMP-9 was purchased from Calbiochem, EMD Biosciences, Inc. (La Jolla, CA), MMP-13 was purchased from R&D Systems, Inc. (Minneapolis, MN), and the synthetic octapeptide MMP substrate (DNP-Pro-Gln-Gly-Ile-Ala-Gly-Gln-dArg) was purchased from Bachem (King of Prussia, PA). Curcumin and CMC 2.5 (1–500 *μ*M) were incubated in 1 mM CaCl_2_, 0.2 M NaCl, and 50 mM Tris/HCl buffer (pH = 7.6) with MMP-9 (gelatinase B) or MMP-13 (collagenase-3) at 37°C for 4 hours as described by us previously [11]. The reaction mixture was quenched with 1,10-phenanthroline (a zinc chelator that binds this cation in the MMP molecule), and the tripeptide degradation fragments of the synthetic octapeptide substrate, after incubation (37°C, l mM Ca^2+^) with each MMP, were measured by high-performance liquid chromatography (HPLC) using a reverse-phase C18 column (4.6 × 75 mm, 3.5 *μ*M macroporous spherical support). The eluate was monitored at 375 nm to quantify the DNP-labeled peptides. The IC_50_ for each compound was calculated from the plot of the percentage of inhibition of enzyme activity versus the concentration of inhibitor [19, 20].

### 2.3. Cell Culture Assay

Human peripheral blood mononuclear cells (PBMC) were isolated and purified from Leukocyte Concentrate (Long Island Blood Bank, Melville, NY) by density gradient centrifugation and adherence using a method described by us previously [21]. PBMC cells were then cultured for 18 hours in serum-free macrophage media (Invitrogen Corp, Carlsbad, CA) at 37°C (95% air, 5% CO_2_) with either LPS derived from *P. gingivalis* (50 ng/mL) or vehicle alone. Curcumin or CMC 2.5 was added at final concentrations of 2 or 5 *μ*M. Conditioned media were analyzed for the cytokines and proinflammatory mediators, TNF-*α*, IL-1*β*, IL-6, MCP-1, and PGE_2_ by ELISA (see below) and for MMP-9 by ELISA and by gelatin zymography (see below) as described previously [21, 22].

### 2.4. Gelatin Zymography

The gelatin zymography system and SDS-PAGE gels, containing polyacrylamide copolymerized with gelatin at a final concentration of 1 mg/mL, were purchased from Invitrogen Corp. (Carlsbad, CA). After electrophoresis (120 V), the gels were washed with 2.5% Triton X-100, incubated at 37°C overnight in calcium assay buffer (40 mM Tris/HCl, 200 mM NaCl, 10 mM CaCl_2_, and pH 7.5), and then stained with Coomassie Brilliant Blue R-250. As described by us earlier [19], clear zones of lysis against a blue background indicate gelatinolytic activity and were scanned densitometrically to assess gelatinase activity. MMP-2 and MMP-9 standards were purchased from R&D Systems, Inc. (Minneapolis, MN).

### 2.5. ELISA Assay

ELISA kits for TNF-*α*, IL-1*β*, IL-6, MCP-1, PGE_2_, and MMP-9 were purchased from R&D Systems, Inc. (Minneapolis, MN). Fifty or 100 *μ*L of the reconstituted standards or samples of conditioned medium was plated into wells coated with anti-human primary antibody and then incubated with 50 *μ*L of a biotinylated detection antibody reagent, at room temperature for two hours. After incubation, the plates were washed three times and 100 *μ*L of streptavidin-HRP solution was added to each well and incubated for 30 minutes at room temperature. Following three further washes, 3,3′, 5,5′′-tetramethylbenzidine (TMB) substrate solution (100 *μ*L) was added to each well and the plate was allowed to develop at room temperature in the dark. After 30 minutes, 100 *μ*L of stop solution was added and the absorbance of the samples was measured at 450 nm [20].

### 2.6. NF*κ*B Activation Assay

The phosphorylation of NF*κ*B was measured by means of a Cellular Activation of Signaling ELISA (CASE) kit (SABiosciences, Frederick, MD). PBMC cells were cultured in serum-free macrophage media (37°C, 95% air, 5% CO_2_) for 18 hours with LPS (*P. gingivalis*, 50 ng/mL) or vehicle alone, and CMC 2.5 was added at a final concentration of 5 *μ*M. Following the incubation, the cells were treated with 4% cell fixing buffer. The wells were washed, quenched, and blocked for 1 hour at 22°C and then incubated with anti-human primary antibodies specific to either phosphorylated or total NF*κ*B protein, for 1 hour at room temperature. After incubation, the plate was washed three times with a buffered surfactant (phosphate buffered saline containing Tween 20), HRP-conjugated secondary antibody solution was added to each well, and the plate was incubated for 60 minutes at room temperature. Following three further washes, a color developing solution was added to each well and the plate was allowed to develop at room temperature in the dark. After 10 minutes, stop solution was added and the absorbance of each sample was measured at 450 nm [23].

### 2.7. Animal Studies

All of the experimental procedures involving animals were approved by Stony Brook University's Institutional Animal Care and Use Committee (IACUC). Twelve male Sprague-Dawley rats (275–300 g body weight; viral antibody free; Charles River Labs) were injected i.v. with streptozotocin (STZ), 70 mg/kg, to induce diabetes and diabetes-enhanced periodontal disease in diabetic rats [14, 15]. Diabetic status was confirmed weekly using a glucose test strip which showed >2% glucose in urine within 24–48 h after STZ injection. Nondiabetic control rats (NDC, *n* = 6) were injected i.v. with the vehicle (citric buffer) alone. One to two days after STZ injection, when glucosuria had been established, six of the STZ-diabetic rats were daily administered for 3 weeks, by oral gavage, a 1 mL suspension of CMC 2.5 (100 mg/kg body weight suspended in 2% carboxymethylcellulose) or 1 mL of vehicle alone (*n* = 6 rats). At the end of the treatment period, the rats were sacrificed by exsanguination, blood samples were collected, and gingiva were dissected and pooled by group, because insufficient gingival tissue is available for individual analysis. Blood glucose levels were analyzed by a blood glucose monitoring system (Johnson and Johnson, Milpitas, CA). Blood samples and gingival tissues were stored at −80°C until analyzed for MMPs and cytokines, by gelatin zymography and ELISA, respectively.

### 2.8. Gingival Extracts

The pooled gingival tissues from each group of rats were weighed, minced (all procedures at 4°C), and extracted with Tris-NaCl-CaCl_2_ buffer (pH 7.6; 100 mg wet weight gingival tissue/5 mL buffer) containing 5 M urea [14, 15]. After centrifugation, the supernatant was dialyzed exhaustively against the Tris/NaCl/CaCl_2_ buffer, and the extract was partially purified by precipitation with ammonium sulfate, added to 60% saturation. Aliquots of each gingival extract were measured for MMP-2 and MMP-9 by gelatin zymography and were scanned densitometrically to quantify gelatinase activity; IL-1*β* was measured using a commercial ELISA kit (R&D systems, Minneapolis, MN).

### 2.9. Western Blot Analysis

Samples were treated with Laemmli buffer (pH 7.0) containing 5 mM dithiothreitol and heated for 5 minutes at 100°C. High- and low-range pre-stained sodium dodecyl sulfate- (SDS-) polyacrylamide gel electrophoresis standard proteins were used as molecular weight markers. The samples were electrophoresed on 7.5% SDS-polyacrylamide gels and then electrophoretically transferred to nitrocellulose membranes. Western blot analysis was carried out as described by us previously [20].

Specific immunoreactivity was visualized as dark bands against a clear background, and the membranes were scanned with an imaging densitometer (Bio-Rad Model GS-700, Bio-Rad, Hercules, CA) using a program (Analyst, Bio-Rad, Hercules, CA) that corrects for background absorption. The densitometric units were measured in the linear range of immunoreactivity for MMP-13; purified human MMP-13 was used as a positive control.

### 2.10. Statistical Analysis

Cytokine and MMP differences in cell culture between groups were analyzed by Student's *t*-test, with *P* ≤ 0.05 taken as statistically significant. In the rat animal studies, when comparing two groups (normal versus untreated diabetic; untreated diabetic versus diabetic treated with CMC 2.5), a student's *t*-test was used as well; *P* values ≤0.05 were considered statistically significant.

## 3. Results

### 3.1. *In Vitro* MMP Inhibition Studies

Each human chromatographically pure MMP was incubated *in vitro* with the collagenase-specific synthetic octapeptide substrate as previously described [20]. For each compound tested as an MMP inhibitor (phenanthroline, curcumin, or CMC 2.5), the concentrations, ranging from 1, 5, 20, to 100 *μ*M, that was required to inhibit 50% of the proteolytic activity of the MMP (IC_50_, see Table 1) was determined from a plot of the extent (%) of inhibition versus the concentration of the inhibitor. 1,10-Phenanthroline, a zinc binding agent [11], was used to quench the *in vitro* MMP assays. Curcumin was used as a positive control. CMC 2.5 was found to inhibit both MMP-9 and MMP-13 activities in a dose-response fashion *in vitro* (data not shown) and was two-seven times more potent (based on IC_50_ values) as an MMP inhibitor than its parent compound, curcumin (Table 1).

### 3.2. Cell Culture Studies

**Figure 1:**
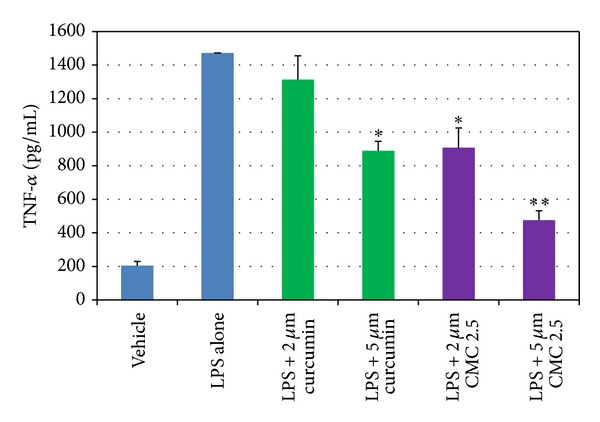
Inhibition of TNF-*α* levels by curcumin and CMC 2.5 in PBMC cells. PBMC cells (5 × 10^5^ cells/well) were cultured in serum-free media (37°C, 95% air, 5% CO_2_) for 18 hours with LPS (*P. gingivalis*, 50 ng/mL) or vehicle alone. Curcumin or CMC 2.5 was added at final concentrations of 2 or 5 *μ*M. Conditioned medium was analysed for TNF-*α* by ELISA. Each value represents the mean of 3 cultures ± the standard error of the mean (S.E.M.). *P < 0.05 represents the significance of all groups of curcumin and CMC 2.5 compared to LPS alone; **P < 0.05 represents the significance between LPS + 5 *μ*M curcumin and LPS + 5 *μ*M CMC 2.5.

**Figure 2:**
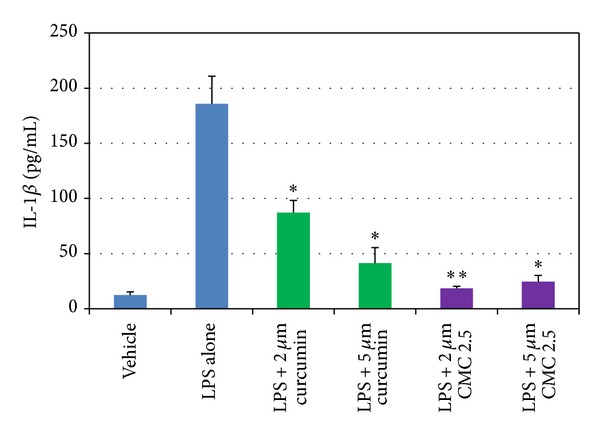
Inhibition of IL-1*β* levels by curcumin and CMC 2.5 in PBMC cells. PBMC cells (5 × 10^5^ cells/well) were cultured in serum-free media (37°C, 95% air, 5% CO_2_) for 18 hours with LPS (*P. gingivalis*, 50 ng/mL) or vehicle alone. Curcumin or CMC 2.5 was added at final concentrations of 2 or 5 *μ*M. Conditioned medium was analysed for IL-1*β* by ELISA. Each value represents the mean of 3 cultures ± S.E.M. *P < 0.05 represents the significance of all groups of curcumin and CMC 2.5 compared to LPS alone; **P < 0.05 represents the significance between LPS + 2 *μ*M curcumin and LPS + 2 *μ*M CMC 2.5.

**Figure 3:**
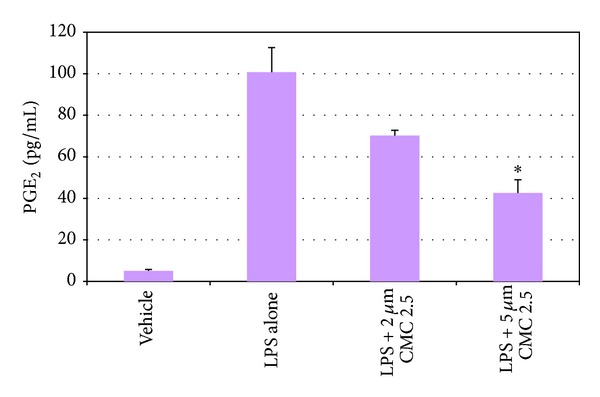
Inhibition of PGE_2_ levels by CMC 2.5 in PBMC cells. PBMC cells (5 × 10^5^ cells/well) were cultured in serum-free media (37°C, 95% air, 5% CO_2_) for 18 hours with LPS (*P. gingivalis*, 50 ng/mL) or vehicle alone. CMC 2.5 was added at final concentrations of 2 or 5 *μ*M. Conditioned medium was analysed for PGE_2_ by ELISA. Each value represents the mean of 3 cultures ± S.E.M. *P < 0.05.

**Figure 4:**
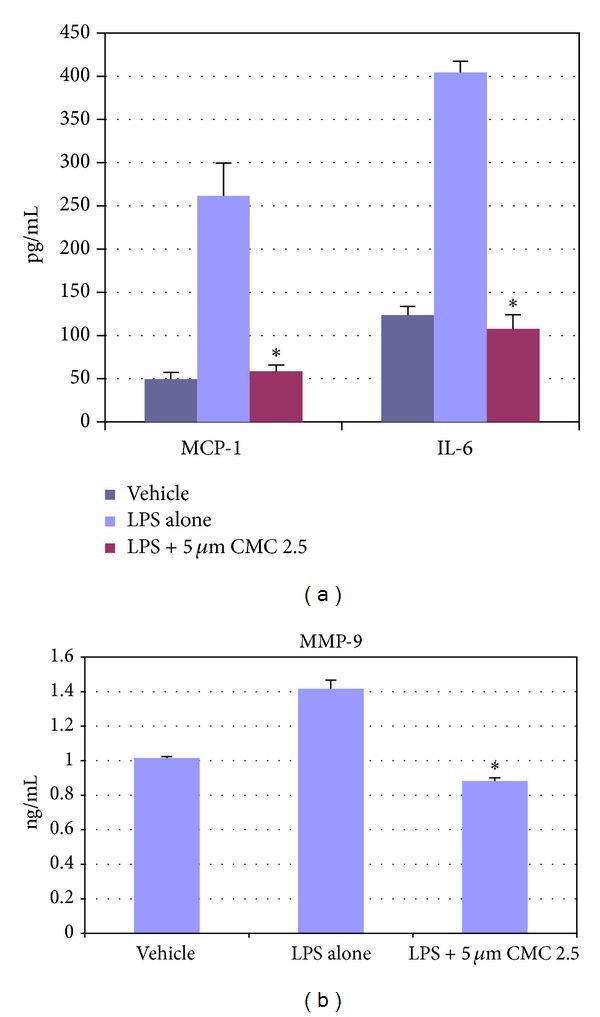
Inhibition of MCP-1, IL-6, and MMP-9 levels by CMC 2.5 in PBMC cells. PBMC cells (5 × 10^5^ cells/well) were cultured in serum-free media (37°C, 95% air, 5% CO_2_) for 18 hours with LPS (*P. gingivalis*, 50 ng/mL) or vehicle alone. CMC 2.5 was added at a final concentration of 5 *μ*M. Conditioned medium was analysed for MCP-1 or IL-6 or MMP-9 by ELISA. Each value represents the mean of 3 cultures ± S.E.M. *P < 0.05.

**Figure 5:**
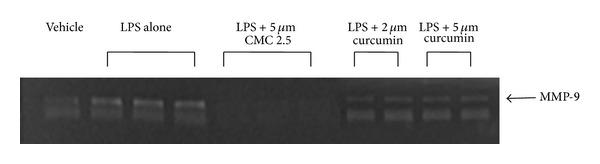
Inhibition of MMP-9 levels by CMC 2.5 in PBMC cells. PBMC cells (5 × 10^5^ cells/well) were cultured in serum-free media (37°C, 95% air, 5% CO_2_) for 18 hours with LPS (*P. gingivalis*, 50 ng/mL) or vehicle alone. CMC 2.5 was added at a final concentration of 5 *μ*M. Conditioned medium was analyzed for MMP-9 by gelatin zymography.

As noted in Figure 1, in our cell culture studies, control wells were incubated with monocytes in serum-free conditioned media (SFCM) (37°C, 95% air, 5% CO_2_) for 18 hours with LPS (*P. gingivalis*, 50 ng/mL) or vehicle alone. In the absence of CMC 2.5 and LPS, about 200 pg/mL of TNF-*α* was secreted by the monocytes which increased to 1470 pg/mL when LPS was added to the culture. TNF-*α* levels were reduced by 13% and 46% with either 2 or 5 *μ*M curcumin, respectively; however, only the latter value was statistically significant (*P* < 0.05). In contrast, when CMC 2.5 was added to the culture of the LPS-stimulated monocytes, in final concentrations of 2 or 5 *μ*M, a greater effect was seen at both concentrations; the extracellular TNF-*α* levels were decreased by 45% (*P* < 0.05) and 79% (*P* < 0.05), respectively. In addition, 5 *μ*M CMC 2.5 was found to be 72% more potent as an inhibitor of TNF-*α* secretion than 5 *μ*M curcumin (*P* < 0.05). Similarly, monocytes secreted 185 pg/mL of IL-1*β* when LPS was added to the culture in contrast to the <25 pg/mL by control cells (Figure 2). IL-1*β* levels were reduced by 57% and 83% with 2 and 5 *μ*M curcumin, respectively (*P* < 0.05). When these cells were incubated in the presence of CMC 2.5 at concentrations of 2 and 5 *μ*M, both concentrations of CMC 2.5 decreased IL-1*β* levels by more than 90%, essentially back to the values seen in cells that were not treated with LPS (Figure 2). All four treatments were statistically significant compared to LPS alone. In fact, 2 *μ*M CMC 2.5 was 75% more effective than 2 *μ*M curcumin (*P* < 0.05) as an inhibitor of IL-1*β* secretion, although the 5 *μ*M CMC 2.5 which appeared to be 50% more effective than 5 *μ*M curcumin did not differ significantly from the effect of 5 *μ*M curcumin (*P* > 0.05). A similar pattern of change was observed for PGE_2_ levels (Figure 3); CMC 2.5 at concentrations of 2 and 5 *μ*M reduced extracellular PGE_2_ levels by 23% (*P* > 0.05) and 51% (*P* < 0.05), respectively, compared to LPS alone. In addition, the extracellular levels of MCP-1, IL-6, and MMP-9 (the latter a major MMP secreted by monocytes) in the SFCM from these monocyte cultures, in the presence or absence of 5 *μ*M CMC 2.5, were analyzed by ELISA and gelatin zymography, respectively. Similar to the results observed for IL-1*β*, the extracellular levels of these bioactive proteins were reduced by more than 90%, essentially down to untreated cell values (Figures 4 and 5) by 5 *μ*M CMC 2.5, and these effects were all statistically significant (*P* < 0.05). In contrast, curcumin at 5 *μ*M concentration did not have a significant effect on MCP-1, IL-6 (data not shown), or MMP-9 levels (Figure 5).

**Figure 6:**
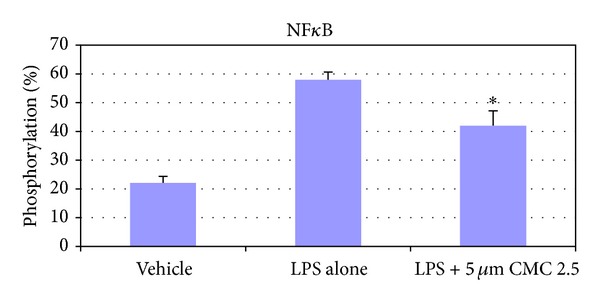
Inhibition of NF*κ*B phosphorylation by CMC 2.5 in PBMC cells. PBMC cells (5 × 10^4^ cells/well) were cultured in serum-free media (37°C, 95% air, 5% CO_2_) for 18 hours with LPS (*P. gingivalis*, 50 ng/mL) or vehicle alone. CMC 2.5 was added at a final concentration of 5 *μ*M. Following the incubation, cells were fixed and phosphorylation of NF*κ*B was analyzed by CASE cellular activation of signaling ELISA kit. *P ≤ 0.05.

To begin to explore the underlying mechanisms of action, the levels of phosphorylation of NF*κ*B (p65/S536) in the presence of CMC 2.5 were analyzed by calculating the percent phosphorylation relative to total NF*κ*B protein levels (Figure 6). In these cultures, control wells were incubated with monocytes in serum-free conditioned media (SFCM; 37°C, 95% air, 5% CO_2_) for 18 hours with LPS (*P. gingivalis*, 50 ng/mL) or vehicle alone. The phosphorylation of NF*κ*B was increased 3-fold in the presence of LPS (Figure 6). When CMC 2.5 was added to the culture of the LPS-stimulated monocytes in a final concentration of 5 *μ*M, the phosphorylation of NF-*κ*B was decreased by 35.4% (*P* ≤ 0.05).

Thus, in these cell culture studies, LPS from gram-negative bacteria, *P. gingivalis*, increased the secretion of TNF-*α*, PGE_2_, IL-1*β*, IL-6, MCP-1, and MMP-9 and increased the phosphorylation (activation) of NF-*κ*B in human mononuclear cells. All of these effects were largely normalized by CMC 2.5. Therefore, the *in vivo* therapeutic potential of this chemically modified curcumin (CMC 2.5) was further evaluated in the diabetes/periodontal inflammation rat model as described below.

### 3.3. *In Vivo* Studies

**Figure 7:**
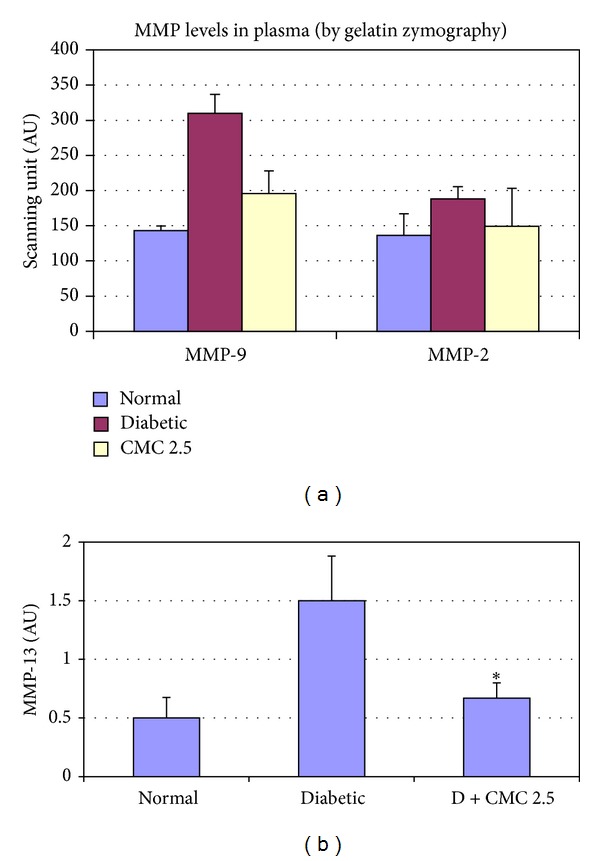
The effect of diabetes and orally administered CMC 2.5 on plasma MMPs. Male Sprague-Dawley rats (*n* = 6) were injected i.v. with streptozotocin (STZ) 70 mg/kg to induce diabetes, as described by us previously. STZ-diabetic rats were daily administered by oral gavage CMC 2.5 (100 mg/kg) for 3 weeks. At the end of the treatment protocol, rats were sacrificed by exsanguination; blood samples were collected and analyzed for MMP-9 by gelatin zymography and MMP-13 by Western blot, and were scanned densitometrically to quantify the levels of MMP-9 and MMP-13. *P < 0.05.

**Figure 8:**
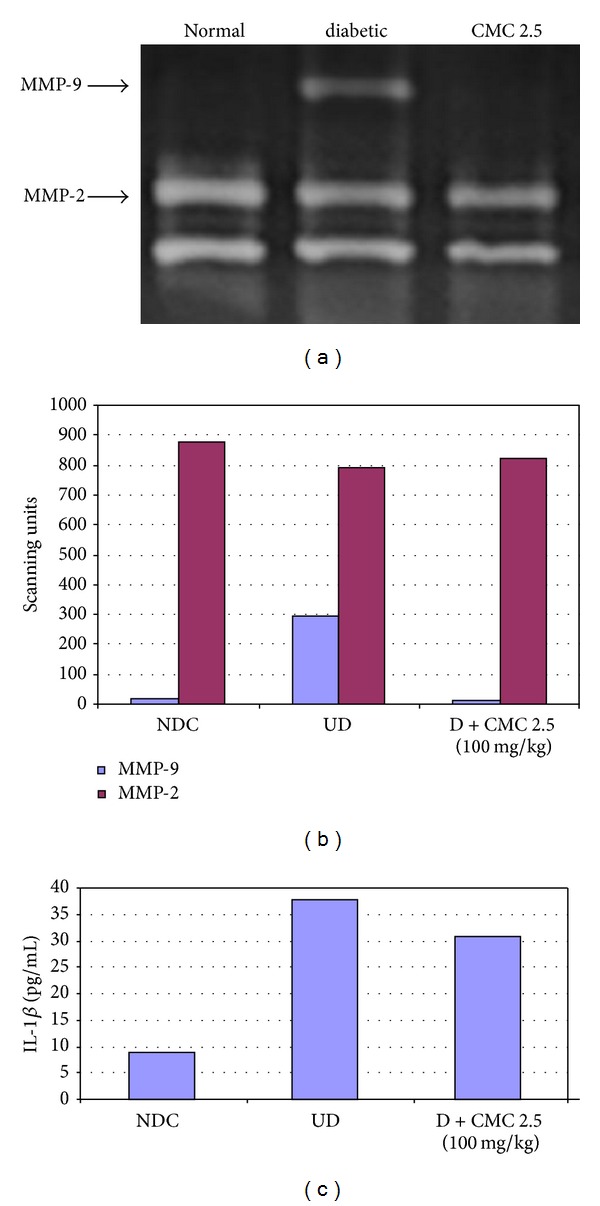
The effect of diabetes and orally administered CMC 2.5 on gingival MMPs (-2, -9) and IL-1*β*. Gingival tissue from diabetic rats treated with vehicle or CMC 2.5 was obtained and pooled by group as described in Section 2, since insufficient gingival tissue is usually available for individual analysis. Gingival tissues were then extracted and aliquots of each gingival extract were measured for MMP-2 and MMP-9 by gelatin zymography and were scanned densitometrically to quantify gelatinase activity, and IL-1*β* was measured by ELISA. NDC: nondiabetic control; UD: untreated diabetic.

**Figure 9:**
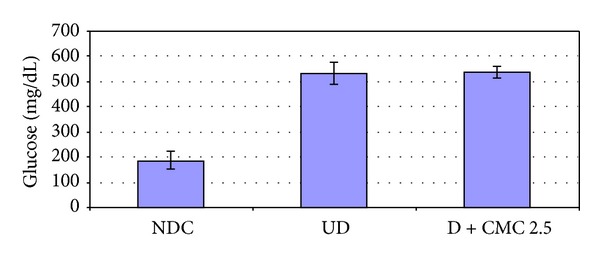
The effect of diabetes and orally administered CMC 2.5 on blood glucose levels. Male Sprague-Dawley rats (*n* = 6) were injected i.v. with streptozotocin (STZ) 70 mg/kg to induce diabetes, as described by us previously. STZ-diabetic rats were daily administered by oral gavage CMC 2.5 (100 mg/kg) for 3 weeks. At the end of the treatment protocol, rats were sacrificed by exsanguination; blood samples were then collected and analyzed for blood glucose levels by the blood glucose monitoring system. NDC: nondiabetic control; UD: untreated diabetic.

The diabetic condition was induced in the rats by STZ as described above. CMC 2.5 (100 mg/kg) was administered orally for 3 weeks beginning after the diabetic condition was established. As expected, we found that the diabetic condition markedly increased the activity of MMP-9 in both plasma and gingiva (Figures 7 and 8). In addition, MMP-13, which in the rats is analogous to MMP-1 in humans [24], was also increased in the plasma of the diabetic rats (Figure 7), whereas MMP-13 was not detected in the pooled gingival tissues. In the plasma, CMC 2.5 reduced the excessive MMP-9 and MMP-13 levels to near normal levels. However, the effects of both diabetes and CMC 2.5 treatment on plasma MMP-2 were not statistically significant, although the pattern of change for this 72 kDa gelatinase paralleled the changes seen for the other MMPs (Figure 7). It should be noted that the elevated blood glucose (Figure 9) and HbA1c (data not shown) levels in the diabetic rats were not affected by CMC 2.5 treatment. In the gingiva, the dominant gelatinase in this periodontal tissue in the nondiabetic control rats is MMP-2, present both as the 72 kDa proform and as the lower molecular weight activated form. Inducing diabetes and severe hyperglycemia results in the induction of MMP-9 (92 kDa gelatinase) in the gingival tissues, but this effect is “normalized” by CMC 2.5 treatment in spite of there being no effect on the severity of the hyperglycemia (Figure 9). Note that the levels/activity of MMP-2 in the gingiva is not affected either by diabetes or by treatment with CMC 2.5. Moreover, the diabetic rats exhibited approximately 200% higher IL-1*β* levels in the gingiva compared to the normal rats, but the oral administration of CMC 2.5 reduced this level in pooled gingival tissue, by 26% (Figure 8).

## 4. Discussion

Curcumin is considered a safe molecule and has been used for thousands of years as a food additive in Asia [25]. Structurally, studies have shown that curcumin contains a *β*-diketone zinc binding site [25] similar to that in the tetracycline-based MMP inhibitors [26]. In the current study, CMC 2.5 demonstrated greater therapeutic activity, compared to curcumin, based on its improved inhibitory activity against MMPs and proinflammatory cytokines in *in vitro* and in cell culture; it also showed efficacy in an animal model of diabetes-enhanced periodontal inflammation (see below). To verify that the inhibition of the MMPs (MMP-9 and MMP-13) and proinflammatory mediators (TNF-*α*, IL-1*β*, MCP-1, IL-6, and PGE_2_) by CMC 2.5 was not the result of cell toxicity, the Cell Proliferation Assay, using a novel tetrazolium compound to determine cell cytotoxicity (MTS assay), was also performed (data not shown). These data demonstrated that CMC 2.5 at doses used in our assay is not toxic to the human mononuclear cells in culture. In additional studies (not shown), no adverse effects were observed in the diabetic rats orally administered doses as high as 500 mg/kg body weight once per day, over a 3-week protocol (much higher than the oral dose used in the current study, 100 mg/kg) [17]. In fact, oral administration of 100 mg/kg CMC 2.5 to the diabetic rats attenuated the complications caused by the severely hyperglycemic condition such as bleeding under the nails, severely inflamed sclera, and impaired wound healing, without any detectable effect on blood glucose or HbA1c levels. This indicates that CMC 2.5 can reduce the destructive effects of the inflammatory mediators (cytokines and MMPs) during local (periodontal disease) and systemic (diabetes) conditions without exhibiting toxicity.

To begin to understand the underlying mechanisms of the inhibitory effects of CMC 2.5 on inflammatory mediators secreted by human mononuclear cells, the phosphorylation of NF-*κ*B was evaluated. Since NF-*κ*B controls transcription of many genes involved in inflammation, it is found chronically activated in many inflammatory diseases such as arthritis [27]. The upregulation of proinflammatory cytokines and MMPs may be mediated through the NF-*κ*B as well as other intracellular signaling transduction pathways including P38 MAP kinase [28]. In the current study, we have shown that LPS stimulation of human monocytes increased, by 3-fold, the phosphorylation of NF*κ*B and that CMC 2.5 can significantly reduce this excessive NF*κ*B activation. Therefore, the increased production of these inflammatory mediators may be regulated, at least partially, through the NF-*κ*B cell signaling transduction pathways, and CMC 2.5 treatment can attenuate this effect.

Diabetes is recognized as an important risk factor for chronic periodontitis based on human clinical trials [29]. The current view is that the hyper- (or prolonged-) inflammatory response during diabetes is mainly caused by the long-term exposure of various proteins to elevated glucose levels. This results in the formation of advanced glycation end-products (AGEs) which promote the secretion of proinflammatory mediators (e.g., TNF-*α*, IL-1*β*, and IL-6) and alters the innate immune response [2, 14], as well as increasing the production and activity of tissue and bone-destructive MMPs [15, 30]. These abnormalities progress to periodontal tissue destruction that is initiated by bacterial factors such as LPS. On the other hand, periodontitis can be more than just a localized chronic inflammation. This oral disease may also have profound effects on the systemic health of the diabetic patient. During the pathogenesis of periodontal disease, the host immunoinflammatory response to plaque bacteria produces destructive cytokines such as TNF-*α*, IL-1*β*, and MMPs [2]. Initially, this response is protective in nature and designed to control the bacterial infection; this can be observed clinically as gingival inflammation with no alveolar bone loss. However, when the above inflammatory process is not well controlled, it results in excessive levels of the inflammatory mediators and MMPs, as seen in diabetes. These inflammatory mediators can eventually enter into the circulation, stimulating a systemic inflammatory response which then increases the risk for developing diabetic complications including an increased risk for cardiovascular disease [2]. In this regard, the interrelationships between diabetes and periodontal disease may represent a “two-hit” model in which diabetes predisposes the patient to oral tissue destruction, and the oral infection exacerbates the abnormal glucose metabolism and its complications in the patient with diabetes [30]. Thus, effective management of both local and systemic inflammation is critical to attenuate these severe complications.

Studies have shown that greater gingival inflammation and periodontal tissue destruction including alveolar bone loss can be observed in diabetic rat models [13–16]. Specifically, in our *in vivo* rat model, we have previously demonstrated [14, 15] that inducing diabetes with streptozotocin (STZ) increases, both systemically (plasma) and locally (gingiva), the levels of cytokines and MMPs which are associated with the collagen and bone destruction that characterize periodontal disease. Alveolar bone loss has been found to be increased in this animal model of diabetes as well [14–16]. Importantly, a number of studies have not found significant differences in periodontal pathogens in the subgingival biofilm in comparing the nondiabetic controls and diabetic humans or rats, indicating that host-response differences between these two groups are largely responsible for the excessively severe periodontal disease in poorly controlled diabetic patients [31]. In the current study, cytokines and MMPs levels were increased, and oral administration of CMC 2.5 to the diabetic rats significantly reduced the MMP-9 and MMP-13 levels in plasma and decreased both MMP-9 and IL-1*β* levels in rat gingival tissue, with no detectable effect on blood glucose or HbA1c levels. However, statistically significant elevated periodontal bone loss in the diabetics was not observed. But, preliminary data in a previous study indicated that CMC 2.5 administration to diabetic rats did significantly reduce alveolar bone loss (*P* < 0.05) [17]. The inconsistency regarding diabetes-induced alveolar bone loss, between the earlier and current studies, may be due to the duration of each study. Changes in inflammatory biomarkers in gingiva presumably occur at an earlier stage while alveolar bone loss takes more time; therefore the latter occurs at a later stage of the disease. In addition, any differences in the age of the rats in the different studies may also play a role in the alveolar bone response to the diabetic condition and drug treatment. Longer term studies, to examine the effects of CMC 2.5 and related congeners on alveolar bone loss in the diabetic rats, are currently underway. In addition, we are also working on a rat model of experimental periodontitis, that is, LPS injection into the gingiva, and have preliminary data showing that CMC 2.5 reduces both MMPs in the gingiva and alveolar bone loss in defleshed jaws in this model. These will be discussed in future reports. Of specific interest, the current study indicates that CMC 2.5 reduces excessive levels of inflammatory cytokines and MMPs in the gingiva and plasma of the diabetic rats at the 3-week time period. This suggests that before the clinical signs of progressive periodontitis are observed, CMC 2.5 can prevent the progression to clinically evident periodontitis (characterized by bone loss), by reducing these inflammatory mediators both locally and systemically at an early stage of the disease. This could also reduce the risk of developing diabetic complications over longer periods of hyperglycemia.

In addition, the *in vitro* MMP inhibition assays were carried out with MMP-9 (92 kDa gelatinase) and MMP-13 (collagenase-3), both known to be associated with periodontal disease and other conditions of connective tissue loss [19, 32]. Our study indicates that CMC 2.5 can inhibit both MMP-9 and MMP-13 activities directly and that CMC 2.5 is more potent as an MMP inhibitor than the parent compound, curcumin. Moreover, the cell culture studies indicate that CMC 2.5 can significantly reduce the MMP-9 and cytokine levels (TNF-*α*, IL-1*β*, MCP-1, IL-6, and PGE_2_) produced by chronic inflammatory cells, in response to the microbial endotoxin, LPS. In addition to monocytes, human polymorphonuclear leukocytes (neutrophils, PMNs) also form an essential part of the innate immune system and play a critical role in acute inflammation. Our studies now underway have shown significant impairment of PMN function in diabetic rats, specifically, reduced PMN chemotaxis and abnormal PMN accumulation in peritoneal exudates from these animals. Moreover, oral administration of a CMC reduced the severity of these abnormalities. These findings will be detailed in future reports [33].

The findings presented in this paper support the hypothesis that a chemically modified curcumin (CMC 2.5) is a pleiotropic compound, having both intracellular and extracellular effects which, collectively, ameliorate local and systemic inflammation and prevent hyperglycemia-associated tissue destruction. Safety and toxicity studies on CMC 2.5 on two animal species, for example, rats and dogs, will be needed to enable proof-of-concept preliminary clinical trials in patients with periodontal disease. Our ultimate goal is to complete additional animal studies and advance to human clinical trials. Future studies using this or related novel CMCs could also test the safety and efficacy of these compounds on animal models of other inflammatory diseases such as rheumatoid arthritis and diabetes-induced impaired wound healing. In conclusion, CMC 2.5, a methoxycarbonyl curcumin, demonstrated therapeutic potential in treating inflammatory and connective tissue-destructive diseases such as periodontal disease and may also reduce the risks of other complications of diabetes.

## Figures and Tables

**Table 1:** Potency of CMC 2.5 as an MMP inhibitor.

Test compounds	MMP-9		MMP-13	
	IC_50_ (*μ*M)	Maximum inhibition (%)	IC_50_ (*μ*M)	Maximum inhibition (%)
1,10-Phenanthroline	9	100	4	100
Curcumin	29	58	110	53
CMC 2.5	16	72	15	69
